# Concurrent Assessment of Phthalates/HEXAMOLL^®^ DINCH Exposure and Wechsler Intelligence Scale for Children Performance in Three European Cohorts of the HBM4EU Aligned Studies

**DOI:** 10.3390/toxics10090538

**Published:** 2022-09-16

**Authors:** Valentina Rosolen, Elisa Giordani, Marika Mariuz, Maria Parpinel, Luca Ronfani, Liza Vecchi Brumatti, Maura Bin, Gemma Calamandrei, Vicente Mustieles, Liese Gilles, Eva Govarts, Kirsten Baken, Laura Rodriguez Martin, Greet Schoeters, Ovnair Sepai, Eva Sovcikova, Lucia Fabelova, Miroslava Šidlovská, Branislav Kolena, Tina Kold Jensen, Hanne Frederiksen, Marike Kolossa-Gehring, Rosa Lange, Petra Apel, Argelia Castano, Marta Esteban López, Griet Jacobs, Stefan Voorspoels, Helena Jurdáková, Renáta Górová, Fabio Barbone

**Affiliations:** 1 Institute for Maternal and Child Health, IRCCS “Burlo Garofolo”, Via dell’Istria 65/1, 34137 Trieste, Italy; 2 Department of Medicine—DAME, University of Udine, Via Colugna 50, 33100 Udine, Italy; 3 Istituto Superiore di Sanità, Viale Regina Elena 299, 00161 Rome, Italy; 4 Center for Biomedical Research, University of Granada, 18011 Granada, Spain; 5 Instituto de Investigación Biosanitaria de Granada, 18012 Granada, Spain; 6 Consortium for Biomedical Research in Epidemiology and Public Health, 28029 Madrid, Spain; 7 VITO Health, Flemish Institute for Technological Research (VITO), 2400 Mol, Belgium; 8 UK Health Security Agency, London SE1 8UG, UK; 9 Department of Environmental Medicine, Faculty of Public Health, Slovak Medical University, 83303 Bratislava, Slovakia; 10 Department of Zoology and Anthropology, Faculty of Natural Sciences and Informatics, Constantine the Philosopher University in Nitra, Nabrezie mladeze 91, 94974 Nitra, Slovakia; 11 Department of Clinical Pharmacology, Pharmacy and Environmental Medicine, Institute of Public Health, University of Southern Denmark, 5000 Odense, Denmark; 12 Department of Growth and Reproduction, Copenhagen University Hospital-Rigshospitalet, 2100 Copenhagen, Denmark; 13 International Center for Research and Research Training in Endocrine Disruption of Male Reproduction and Child Health (EDMaRC), Copenhagen University Hospital-Rigshospitalet, 2100 Copenhagen, Denmark; 14 German Environment Agency (UBA), Corrensplatz 1, 14195 Berlin, Germany; 15 National Centre for Environmental Health, Instituto de Salud Carlos III, 28029 Madrid, Spain; 16 Unit Separation and Conversion Technology, Flemish Institute for Technological Research (VITO), Boeretang 200, 2400 Mol, Belgium; 17 Department of Analytical Chemistry, Faculty of Natural Sciences, Comenius University, Ilkovičova 6, Mlynská Dolina, 84215 Bratislava, Slovakia; 18 Institute of Hygiene and Clinical Epidemiology, Azienda Sanitaria Universitaria Friuli Centrale, Via Colugna 50, 33100 Udine, Italy

**Keywords:** human biomonitoring, children, phthalates, HEXAMOLL^®^ DINCH, neurodevelopment, WISC, Full-Scale Intelligence Quotient, environmental contaminants, aligned studies, HBM4EU

## Abstract

Information about the effects of phthalates and non-phthalate substitute cyclohexane-1,2-dicarboxylic acid diisononyl ester (HEXAMOLL^®^ DINCH) on children’s neurodevelopment is limited. The aim of the present research is to evaluate the association between phthalate/HEXAMOLL^®^ DINCH exposure and child neurodevelopment in three European cohorts involved in HBM4EU Aligned Studies. Participating subjects were school-aged children belonging to the Northern Adriatic cohort II (NAC-II), Italy, Odense Child Cohort (OCC), Denmark, and PCB cohort, Slovakia. In each cohort, children’s neurodevelopment was assessed through the Full-Scale Intelligence Quotient score (FSIQ) of the Wechsler Intelligence Scale of Children test using three different editions. The children’s urine samples, collected for one point in time concurrently with the neurodevelopmental evaluation, were analyzed for several phthalates/HEXAMOLL^®^ DINCH biomarkers. The relation between phthalates/HEXAMOLL^®^ DINCH and FSIQ was explored by applying separate multiple linear regressions in each cohort. The means and standard deviations of FSIQ were 109 ± 11 (NAC-II), 98 ± 12 (OCC), and 81 ± 15 (PCB cohort). In NAC-II, direct associations between FSIQ and DEHP’s biomarkers were found: 5OH-MEHP+5oxo-MEHP (β = 2.56; 95% CI 0.58–4.55; N = 270), 5OH-MEHP+5cx-MEPP (β = 2.48; 95% CI 0.47–4.49; N = 270) and 5OH-MEHP (β = 2.58; 95% CI 0.65–4.51; N = 270). On the contrary, in the OCC the relation between DEHP’s biomarkers and FSIQ tended to be inverse but imprecise (*p*-value ≥ 0.10). No associations were found in the PCB cohort. FSIQ was not associated with HEXAMOLL^®^ DINCH in any cohort. In conclusion, these results do not provide evidence of an association between concurrent phthalate/DINCHHEXAMOLLR DINCH exposure and IQ in children.

## 1. Introduction

Evidence of the presence of environmental contaminants in human samples is increasing. Phthalates and their substitute cyclohexane-1,2-dicarboxylic acid diisononyl ester (HEXAMOLL^®^ DINCH), a group of non-persistent chemicals [1,2], have been found ubiquitously [3,4,5,6,7,8] in human samples due to their common use as plasticizers in polymers like polyvinylchloride (PVC) resins, components of personal care products, constituents of food contact materials and medical devices [2,9,10,11,12]. Human exposure to the substitute HEXAMOLL^®^ DINCH has increased significantly in the last years [13,14,15] and has been shown to have disruptive thyroid system properties in pregnant women [16].

Routes of exposure to phthalates and HEXAMOLL^®^ DINCH are usually ingestion, inhalation, and dermal contact [2,17]. Exposure to high molecular weight (HMW) phthalates, including 2-ethylhexyl phthalate (DEHP), is primarily related to food consumption [18,19,20,21] since these compounds are not chemically bound to food contact materials and can leach into packaged foods. Low molecular weight (LMW) phthalates such as diethyl phthalate (DEP), butylbenzyl phthalate (BBzP), di-n-butyl phthalate (DnBP), and di-iso-butyl phthalate (DiBP) are commonly found in personal care products and house dust [20,22,23,24]. Phthalates and HEXAMOLL^®^ DINCH urinary metabolites (either oxidized or not monoesters) have been considered the most suitable biomarkers of exposure [2,25,26,27].

Except in some cases of DEP exposure [3,23,28,29,30], vulnerable subgroups of the population, especially children, have been found to have higher urinary levels of phthalates and HEXAMOLL^®^ DINCH biomarkers than adults and adolescents [3,4,6,7,14,28,31], due to their different phthalate oxidative metabolism [2], uptake patterns (hand-to-mouth behavior), mouthing of plastic toys [19,32,33], chewing plastic child products, and lower body weight [4,24,28,34,35]. According to the hypothesis of the developmental origin of health and diseases [36], an increasing number of scientific observations have shown that exposure to environmental contaminants during pregnancy, infancy, and early childhood could be a risk factor for diseases that are diagnosed in adulthood.

Due to their reprotoxic and endocrine-disrupting properties, the commercialization in the European Union of DEHP, BBzP, DiBP, and DnBP is strictly regulated. The activation of nuclear receptors like peroxisome proliferator-activated receptors (PPARα and PPARγ) and glucocorticoid receptors by phthalates may initiate events leading to several adverse health outcomes [37].

Epidemiological studies on the association between pre- and/or postnatal phthalate exposure and health effects are increasingly calling attention to issues related to reproductive function, allergies and asthma, cardiometabolic diseases, and thyroid function [38,39,40,41,42].

Some epidemiological investigations and reviews have also shown positive associations between pre- and/or postnatal exposure and adverse neurodevelopmental function [43,44,45,46]. However, the overall evidence on the association between phthalates/HEXAMOLL^®^ DINCH metabolites and child neurodevelopment is limited. Lee et al. [47] showed an inverse association between postnatal exposure to DEHP and cognitive function. Indeed, cognitive functions together with several brain areas continue to develop during childhood [48]. Sex-specific associations were reported between BBzP and decreased motor abilities in females [49]. It is worth noting that only a few cohorts have been primarily established to study the association between phthalate metabolites and neurodevelopmental outcomes, and studies on the links between HEXAMOLL^®^ DINCH and neurological development in children have not been conducted yet.

In 2016 the “European Human Biomonitoring Initiative” (HBM4EU) [50] was launched in 30 countries and the European Environment Agency with the aim to improve chemical safety, creating a European network that improves knowledge thanks to harmonization, planning, and implementation of human biomonitoring (HBM) studies, as well as harmonized sampling and data analysis across national borders. Plasticizers, like phthalates and their substituent HEXAMOLL^®^ DINCH, have been identified by European Union (EU) services and HBM4EU partners as priority substances for chemical policy at the EU level [51], for which open policy-relevant questions still had to be answered.

This research aims to evaluate the association between child neurodevelopment, measured by the Wechsler Intelligence Scale for Children (WISC), and phthalate/HEXAMOLL^®^ DINCH exposure measured in children’s urine in three European cohorts involved in the HBM4EU Aligned Studies [52,53]. The latter is a survey aimed at collecting HBM samples and data as harmonized as possible from national studies to derive current internal exposure data or the European population/citizens across a geographic spread.

## 2. Materials and Methods

### 2.1. Data Source

European countries involved in the HBM4EU Aligned Studies that provided data on the assessment for child neurodevelopment and the measurement of phthalates and HEXAMOLL^®^ DINCH exposure were: Italy (Department of Medicine, University of Udine: Northern Adriatic Cohort II (NAC-II)) [54,55], Denmark (Odense University Hospital, Odense Child Cohort (OCC)) [56,57], and Slovakia (Department of Environmental Medicine, Faculty of Public Health, Slovak Medical University: PCB cohort) [58,59]. Participating subjects were 7-year-old children belonging to the NAC-II and OCC and 11-year-old children in the PCB cohort. The total number of subjects in the original studies was 487 for the NAC-II, 2449 for the OCC, and 415 for the PCB cohort. The selection of the 300 participants of each cohort was performed following a step-wise selection procedure described in Gilles et al., 2022 [53].

Informed consent was obtained from all participant’s caregivers involved in the study. The study was conducted according to the Declaration of Helsinki. Research protocols of the three cohorts were approved by their respective ethics committees (NAC-II: Ethics Committee of the Institute for Maternal and Child Health IRCCS “Burlo Garofolo” (CE/V-70-05/02/2007; CE/V-109-12/04/2010; IRB-BURLO 01/2020 25/03/2020); PCB cohort: Ethics Committee of the Slovak Medical University from 18 November 2013; OCC: Regional Scientific Ethical Review Committee for Southern Denmark (Project ID S-20090130) and the Danish Data Protection Agency (j.no. 18/33119)).

### 2.2. Study Outcome

In the three cohorts, the neurodevelopment of children was assessed by trained psychologists using the WISC test [60]. Different editions of the WISC test were used in the three cohorts: the 3rd in the PCB cohort [61], the 4th in the NAC-II [62], and the 5th in the OCC [63]. The three editions of the WISC test assess and measure different aspects of the child’s neurodevelopment providing for each edition different index scores, which when combined, yield the Full-Scale Intelligence Quotient (FSIQ). A brief description of scores obtained from the three editions of WISC and of the method of computation is included in the Supplemental Materials (Table S1).

We considered the outcome as the measure of the child’s overall cognitive ability, i.e., the FSIQ score. The FSIQ score ranged from 40 to 160 and was set to have a mean of 100 and a standard deviation of 15 based on Italian, Slovak, or Danish population-based reference data (standard population). A higher FSIQ score should be interpreted as a better child’s neurodevelopment performance, although no clinical diagnosis can be made according to any low or very low FSIQ score.

### 2.3. Exposure

Concurrently with the outcome evaluation, exposure assessment was conducted by analyzing DiBP, DnBP, BBzP, DEP, DEHP, and HEXAMOLL^®^ DINCH metabolites in urinary samples. The following biomarkers of exposure were measured in the children’s urine: mono-isobutyl phthalate (MiBP) for DiBP, mono-n-butyl phthalate (MnBP) for DnBP, mono-benzyl phthalate (MBzP) for BBzP, mono-ethyl phthalate (MEP) for DEP, mono(2-ethyl-5-hydroxy-hexyl) phthalate (5OH-MEHP), mono(2-ethyl-5-carboxy-pentyl) phthalate (5cx-MEPP) and mono(2-ethyl-5-oxo-hexyl) phthalate (5oxo-MEHP) for DEHP and cyclohexane-1,2-dicarboxylate-mono-(7-hydroxy-4-methyl)octyl ester (OH-MINCH), and cyclohexane-1,2-dicarboxylate-mono-(7-carboxylate-4-methyl)heptyl ester (cx-MINCH) for HEXAMOLL^®^ DINCH.

### 2.4. Chemical Analysis

In the NAC-II between 2014 and 2016, 7-year-old children’s spot urine samples were collected in a 50 mL tube (BD Falcon) and stored at −80 °C within 24 h of collection.

In the OCC between 2018 and 2019, 7-year-old children’s spot urine samples were collected and stored at −80 °C until chemical analyses.

In the PCB cohort between 2014 and 2017, 11-year-old children’s spot urine samples were collected in 50 mL polypropylene tubes and stored at −18 °C.

Urinary concentrations of phthalates (except for PCB cohort) and HEXAMOLL^®^ DINCH biomarkers were analyzed under the Quality Control/Quality Assurance (QA/QC) program developed within HBM4EU [64,65] following protocols HBM4EU-SOP-QA-001 to 004 which are available through the HBM4EU website [50]. External quality control was assured through successful participation in the External Quality Assessment Scheme (EQUAS) for phthalates biomarkers in urine, organized by the Institute and Outpatient Clinic of Occupational, Social and Environmental Medicine (IPASUM), Germany. EQUAS was organized within the frame of HBM4EU as part of the Quality Assurance program for biomonitoring analyses.

Urinary concentrations of phthalates biomarkers in the PCB cohort were analyzed under the method certified by the quality assurance program (Intercomparison program 57, 2016 for occupational/environmental medical—toxicological analyses in biological materials) organized by IPASUM.

#### 2.4.1. Phthalates

All samples were analyzed in laboratories that participated and obtained successful results in the HBM4EU program [64,65].

VITO—GOAL Laboratories (Belgium) analyzed the NAC-II urinary samples. After defrosting, NAC-II urine samples were vortexed and sonicated for 5 min and then analyzed following the methodology described by Servaes et al. [66]. Briefly, β-glucuronidase (from *Escherichia coli* K-12) and 50 µL of corresponding internal standards (d_4_ and ^13^C isotope labeled analogues for phthalate biomarkers) were added to 1 mL of sample buffered with sodium acetate solution. After incubation at 37 °C for at least 2 h, 10 µL of the solution was injected into an ultra-performance liquid chromatography-tandem mass spectrometer (UPLC-MS) to detect the above-mentioned phthalate biomarkers. The phthalates’ biomarkers were separated on an Acquity UPLC BEH phenyl column (100 mm × 2.1 mm; 1.7 µm) coupled to a Waters Xevo TQ-S tandem mass spectrometer, operating in the negative electrospray ionization mode (ESI−).

The OCC’s urine sample analyses were conducted at the Department of Growth and Reproduction, Copenhagen University Hospital—Rigshospitalet, Denmark (RegionH). The phthalate biomarkers were analyzed by isotope diluted online-TurboFlow liquid chromatography mass spectrometry (LC-MS/MS) equipped with a probe for heated electrospray ionization (HESI) running in negative mode and with prior enzymatic deconjugation. The preceding enzymatic deconjugation of the biomarkers was done by arylsulfatase-free β-glucuronidase (*Escherichia coli* K12). A detailed description of the analytical method has previously been published [15]. Briefly, samples were analyzed in nine batches, all including standards for calibration curves; three blanks, three un-spiked urine pool controls, and three urine pool controls spiked with a mixture of native phthalate metabolite standards in low and high concentration levels. The mean recovery was >88% for all biomarkers in urine pool controls spiked in both levels, while the mean relative standard deviation (RSD) was <11% for all biomarkers in urine controls spiked at a low level, except for MEP, MiBP, and MnBP (<18%), and <6% for all biomarkers in urine controls spiked at high level.

Phthalates’ urinary biomarkers of the PCB cohort were measured in the Physiological analytical laboratory Constantine the Philosopher University in Nitra, Slovakia. Urinary concentration was determined by high-performance liquid chromatography (HPLC) and tandem mass spectrometry.

Briefly, 1 mL of urine was thawed, buffered with ammonium acetate, spiked with isotope-labeled phthalate standards, β-glucuronidase enzyme, and incubated at 37 °C. The samples were then diluted with phosphate buffer (NaH_2_PO_4_ in H_3_PO_4_) and loaded on solid-phase extraction (SPE) cartridges conditioned with acetonitrile followed by phosphate buffer before extraction. To remove the hydrophilic compound, SPE cartridges were flushed by formic acid and HPLC grade water. Elution of analytes was performed by acetonitrile and ethylacetate. Eluate was dried with nitrogen gas and reconstituted with 200 µL of H_2_O and acetonitrile (1:1). An Agilent Infinity 1260 liquid chromatograph equipped with EC 150 × 3 Nucleodur phenyl-hexyl columns was used for analysis. Separation was done using a nonlinear gradient program of two mobile phases (acetonitrile and 0.1% acetic acid in H_2_O). Agilent 6410 triplequad with electrospray ionization was used in the negative mode for mass-specific detection of phthalate biomarkers. RSD was not higher than 11% for all biomarkers in urine pool controls.

#### 2.4.2. HEXAMOLL^®^ DINCH

In the OCC, urine samples were analyzed at RegionH laboratories, while in the NAC-II and PCB cohort, they were analyzed at the Department of Analytical Chemistry, Faculty of Natural Sciences, Comenius University in Bratislava, Slovakia (UNIBA).

UNIBA determined cx-MINCH and OH-MINCH urinary concentrations after enzymatic hydrolysis of their glucuronide conjugates by high-performance liquid chromatography-tandem mass spectrometry (HPLC-MS/MS) with online SPE. Briefly, 300 µL aliquots of urine samples were thawed and vortexed. Subsequently, 100 µL of ammonium acetate buffer (pH 6), 10 µL of internal standards solution (d_8_ isotope labeled analogues for HEXAMOLL^®^ DINCH biomarkers, at a concentration of 2 mg/L) and 6 µL of β-glucuronidase solution (from *Escherichia coli* K-12) were added. After vortexing and incubation at 37 °C for 2 h, 10 µL of acetic acid was added and samples were frozen overnight (−20 °C) to cryoprecipitate the proteins. After thawing and centrifugation (10 min, 10,000 rpm), 210 µL supernatant was injected into a C-18 SPE column (20 mm × 4.6 mm, 3.5 µm) at isocratic elution of 90% mobile phase A (water with 0.05% acetic acid) and 10% B (acetonitrile with 0.05% acetic acid) for 5 min, then the flow was switched to the analytical column. Separation was performed using Zorbax-SB C-18 column (150 mm × 2.1 mm; 3.5 µm), and gradient elution from 10% to 100% B. Thermo Fisher Scientific TSQ triple quadrupole mass spectrometer with a heated electrospray ionization source operated in negative mode was used for measurements.

RegionH determined cx-MINCH and OH-MINCH concentrations in urine samples by applying the same chemical analytical process described above for the phthalate biomarkers.

#### 2.4.3. Creatinine

To account for urinary dilution, creatinine analysis was performed.

In NAC-II and PCB cohorts, UNIBA determined the creatinine concentration (µg/L) by flow injection analysis-tandem mass spectrometry (FIA-MS/MS). In the OCC, spectrophotometric determination of creatinine concentrations was conducted on a Konelab 20 Clinical Chemistry Analyzer, using a commercial kit (Thermo, Vantaa, Finland) at the University of Southern Denmark (SDU) in the Environmental Medicine Laboratory.

### 2.5. Potential Confounders

Based on information obtained from the questionnaires applied in the different aligned studies, variables were harmonized across the studies and data/variables needed for our research were shared with the principal investigators of this manuscript [52,53].

Potential confounders in neurodevelopment were found through a literature review, and those common to the three cohorts were the following: the highest education level of the household of the child, body mass index (BMI) z-score, and sex of the child. The level of education of the parents was based on the International Standard Classification of Education (ISCED) developed by the United Nations Educational, Scientific, and Cultural Organization (UNESCO) [67]. Individuals with no to lower secondary education are included in the lower education level (ISCED 0–2), individuals with upper secondary to post-secondary non-tertiary education represent the medium level (ISCED 3–4), and those with tertiary and higher education are considered into the high education level (ISCED ≥5). The highest education level of the household of the child corresponds to the higher level of education between mother and father.

At urine sample collection, children’s height and weight were measured by healthcare staff in each cohort. BMI (kg/m^2^) was calculated as the ratio between the weight (kg) and the height squared (m^2^). BMI was converted into standardized World Health Organization sex- and age-specific z-scores (BMI z-score) [68].

### 2.6. Statistical Analysis

The outcome variable (FSIQ score) was obtained from three different versions of the WISC test, and for this reason, separate statistical analyses were conducted for each cohort.

To account for the fact that the study subjects belong to three different cohorts, where three different versions of the WISC were used, we performed multilevel fixed-effect linear regression analyses, adjusted for the highest education level of the household of the child, child’s sex and BMI z-score. The Intraclass Correlation Coefficient (ICC) was estimated and indicates how much of the total variation in FSIQ is accounted for by the cohorts. The Akaike Information Criterion (AIC) was used to evaluate the model fitting and to select the most appropriate model. Beta coefficient (β), 95% confidence interval (95% CI), and ICC were reported. Separate models were applied for each biomarker.

In each cohort, the main characteristics of children and their families were described as frequencies and percentages for categorical variables or as arithmetic means and standard deviations (SD) for continuous variables.

The FSIQ score and creatinine concentrations were described as the arithmetic mean and SD, median, 25th and 75th percentile. For phthalates and HEXAMOLL^®^ DINCH biomarker concentrations, the geometric mean and 95% CI, and 90th percentile were also calculated (the Supplementary Materials also show the biomarker’s concentration standardized for creatinine). Differences among cohorts for the highest education level of the household of the child were assessed by the Chi-square test.

The difference in the means of FSIQ score among the three cohorts was assessed by applying ANOVA with Bonferroni’s multiple comparison method.

Among biomarkers, several sum-parameters were determined, summing the single biomarker’s concentrations as follows: 5OH-MEHP+5cx-MEPP and 5OH-MEHP+5oxo-MEHP for DEHP, and OH-MINCH+cx-MINCH for HEXAMOLL^®^ DINCH.

Pearson correlations between the FSIQ score and biomarkers (data not shown) and BMI were calculated. ANOVA was applied to assess the mean differences in the FSIQ score among the highest education level of the household of child and child’s sex.

The relations between the FSIQ score and each phthalate/HEXAMOLL^®^ DINCH biomarker were explored, applying simple and multiple linear regressions adjusted for the highest education level of the household of the child, child’s sex, and BMI z-score. β and 95% CI of each multiple linear regression were presented in the forest plot grouped by phthalates and HEXAMOLL^®^ DINCH (in the Supplementary Material; Figures S2–S4 show the forest plots grouped by cohort). Separate models were applied for each biomarker in each cohort. Phthalates and HEXAMOLL^®^ DINCH biomarker concentrations were natural logarithm transformed because of their skewed distribution and standardized according to child urine’s creatinine levels (µg/g creatinine) to account for urinary dilution. As sensitivity analyses, simple and multiple regression models were performed considering the natural logarithm transformation of phthalates and HEXAMOLL^®^ DINCH biomarker concentrations without the creatinine standardization and the natural logarithm transformation of the biomarkers 5OH-MEHP+5cx-MEPP, 5OH-MEHP+5oxo-MEHP, and OH-MINCH+cx-MINCH expressed in molar unit standardized for creatinine.

SAS (version 9.4 SAS Institute Inc., Cary, NC, USA) and R software (package “anthroplus” in R; R version 4.0.5, R Core Team (2021). R: A language and environment for statistical computing. R Foundation for Statistical Computing, Vienna, Austria. URL https://www.R-project.org/ (accessed on 30 June 2022) were used for the statistical analysis.

## 3. Results

For each cohort (NAC-II, OCC, and PCB cohorts), 300 children participated in the study. Child and family characteristics of the three cohorts are reported in Table 1. Mean values and SD of children’s age were 7.0 ± 0.2 in the NAC-II, 7.0 ± 0.2 in the OCC, and 11.1 ± 0.4 in the PCB cohort. The percentage distribution of the highest education level of the household of the child were different among the three cohorts. The percentage of children from a household with high education level was equal to 44.0%, 35.0%, and 15.0%, respectively, in the NAC-II, OCC, and PCB cohort (Chi-square test *p*-value ≤ 0.05).

**Figure 1:**
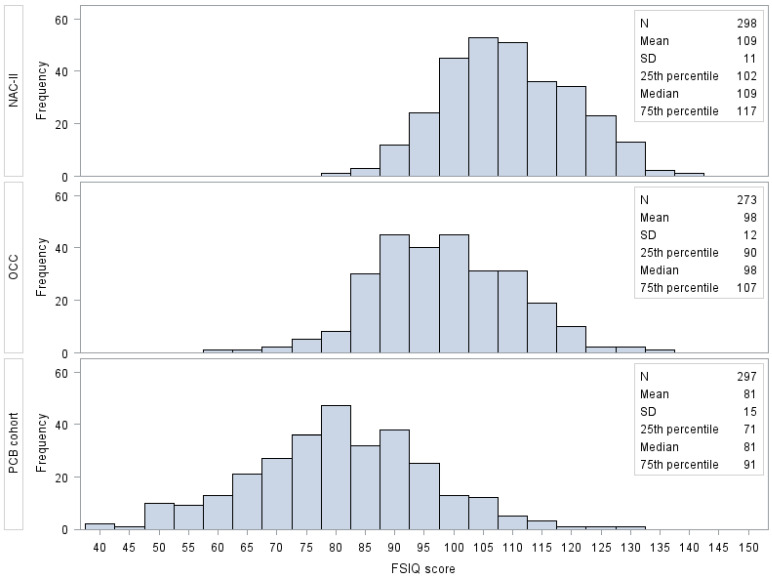
Frequency distribution of FSIQ score by cohort. Different WISC editions were used among the cohorts: the 3rd in the PCB cohort, the 4th in the NAC-II, and the 5th in the OCC. Abbreviations: FSIQ, Full-Scale Intelligence Quotient; NAC-II, Northern Adriatic Cohort; OCC, Odense Child Cohort; SD, standard deviation.

The distribution of the FSIQ score is shown in Figure 1. The difference in means of the FSIQ score among the three cohorts performing Bonferroni’s multiple comparisons method were all statistically significant (*p*-value ≤ 0.05).

Urinary concentrations of phthalates and HEXAMOLL^®^ DINCH biomarkers for each cohort are reported in Table 2.

Urinary concentrations of phthalates and HEXAMOLL^®^ DINCH biomarkers expressed as µg/g creatinine and creatinine concentrations (g/L) for all cohorts are reported in Supplementary Table S2.

As displayed in Table 3, in each cohort, the FSIQ score increased with the increasing education level of the household of the child, whereas the means of FSIQ score did not vary by child’s sex (*p*-value ≤ 0.05). The FSIQ score was directly correlated with BMI z-score in the PCB cohort (r = 0.16, *p*-value ≤ 0.05), inversely correlated in the NAC-II (r = −0.14, *p*-value ≤ 0.05) and not correlated in the OCC cohort (r = 0.03, *p*-value = 0.56).

**Figure 2:**
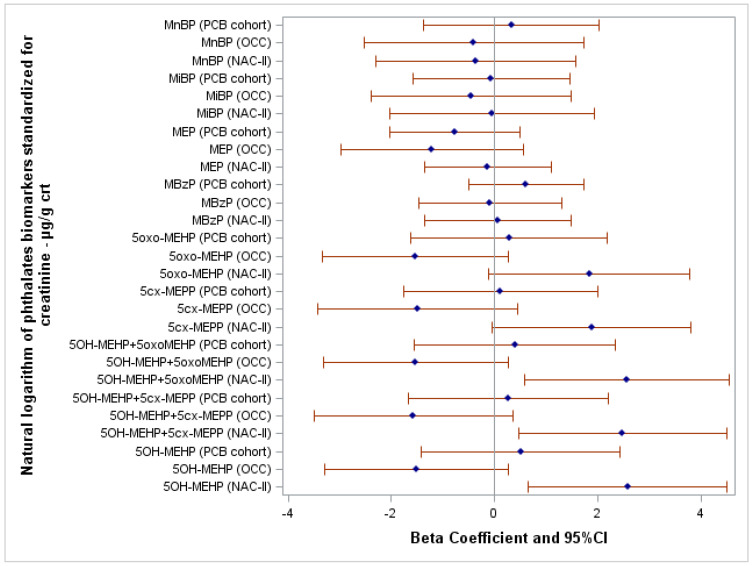
Forest plot showing the association between FSIQ score and the natural logarithm of phthalates biomarkers standardized for creatinine adjusted for the highest education level of the household of the child, child’s sex, and body mass index z-score. Abbreviations: crt, creatinine.

The results of the multilevel fixed-effect linear regression models are shown in Table S3. Figure 2 shows the association between the FSIQ score and the natural logarithm of phthalates biomarkers standardized for creatinine and adjusted for potential confounders. Crude and adjusted β and 95% CI of all linear regression models are reported in Supplementary Table S4. Among NAC-II children, FSIQ scores were directly associated with the following DEHP biomarkers: 5OH-MEHP (β = 2.58; 95% CI 0.65–4.51), 5OH-MEHP+5oxo-MEHP (β = 2.56; 95% CI 0.58–4.55), and 5OH-MEHP+5cx-MEPP (β = 2.48; 95% CI 0.47–4.49). Moreover, FSIQ score increased, albeit not significantly, at somewhat higher concentrations of 5oxo-MEHP (β = 1.83; 95% CI −0.11–3.78; *p*-value = 0.06) and 5cx-MEPP (β = 1.88; 95% CI −0.04–3.80; *p*-value = 0.06).

In the OCC cohort, associations tend to be inverse, but imprecise, between FSIQ score and 5OH-MEHP (β = −1.51; 95% CI −3.29–0.27; *p*-value = 0.10), 5OH-MEHP+5oxo-MEHP (β = −1.53; 95% CI −3.32–0.27; *p*-value = 0.10), 5OH-MEHP+5cx-MEPP (β = −1.58; 95% CI −3.50–0.35; *p*-value = 0.11), 5oxo-MEHP (β = −1.53; 95% CI −3.33–0.27; *p*-value = 0.10), and 5cx-MEPP (β = −1.49; 95% CI −3.43–0.45; *p*-value = 0.13). In the PCB cohort, no significant associations were found for the same biomarkers.

No significant associations were found between the FSIQ score and the remaining biomarkers of phthalates.

**Figure 3:**
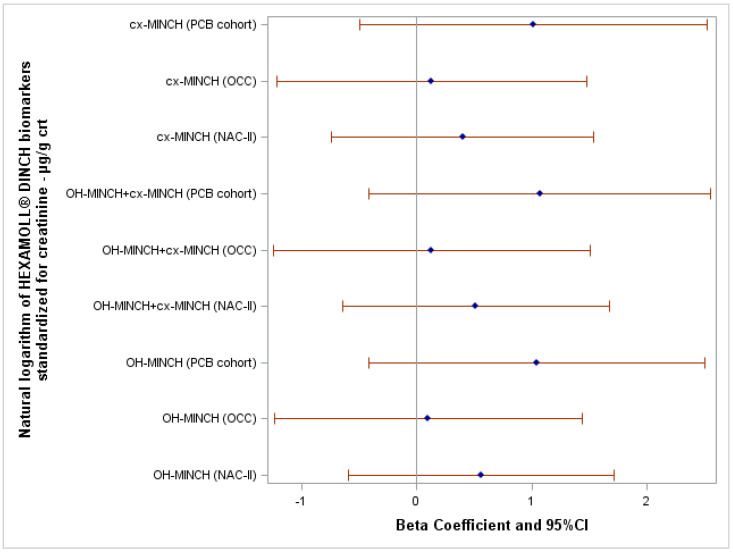
Forest plot showing the association between FSIQ score and the natural logarithm of HEXAMOLL^®^ DINCH biomarkers standardized for creatinine adjusted for the highest education level of the household of the child, child’s sex, and body mass index z-score.

After adjusting for potential confounders, the natural logarithm of HEXAMOLL^®^ DINCH biomarkers standardized for creatinine were not associated with the FSIQ score in the three cohorts (Figure 3). Crude and adjusted β and 95% CI of all linear regression models are reported in Supplementary Table S4.

Supplementary Table S5 reports the results of simple and multiple linear regression between the FSIQ score and the natural logarithm transformation of phthalates/HEXAMOLL^®^ DINCH biomarkers not standardized for creatinine.

The results of simple and multiple linear regression models between the FSIQ score and the natural logarithm transformation of the biomarkers 5OH-MEHP+5cx-MEPP, 5OH-MEHP+5oxo-MEHP, and OH-MINCH+cx-MINCH expressed in molar unit standardized for creatinine in the NAC-II, OCC, and PCB cohorts are shown in Table S6.

## 4. Discussion

### 4.1. Internal Consistency

The study protocol hypothesized that increased levels of biomarkers of phthalates and HEXAMOLL^®^ DINCH, adjusted for confounders, might be associated with a decreased child’s overall cognitive ability. Contrary to this hypothesis, the results of this evaluation lack significant and consistent evidence of inverse associations between this group of exposures and FSIQ. In addition, as described in Figure 2 and Figure 3, results varied widely by type of phthalates and HEXAMOLL^®^ DINCH metabolites. Results related to MnBP, MiBP, and MBzP, and HEXAMOLL^®^ DINCH biomarkers do not show significant associations in either direction, and β estimates do not differ greatly by cohort. By contrast, results on DEHP phthalates (5OH-MEHP, 5oxo-MEHP, and 5cx-MEPP and their sum-parameters) show opposite directions of the association by cohort. In fact, linear regression models display direct associations between child’s FSIQ score and some DEHP biomarkers in the Italian and Slovak cohorts, although the relation was not statistically significant in the latter. Whereas in the Danish OCC, the associations between the FSIQ score and the DEHP biomarkers tend to be inverse albeit statistically imprecise (*p*-value ≥ 0.1). At the same time, DEHP exposure in Danish children averages two to four times lower than in Italian children. However, at increasing levels of DEHP biomarkers, Italian children perform *better* according to FSIQ. These inconsistent results, especially those pertaining to the group of DEHP biomarkers, elicit several potential interpretations. According to an inverse, linear relation, should phthalates exposure be a cause of lower IQ scores in children, we would expect to see it more clearly in the cohorts that are exposed to higher levels, not otherwise. To reconcile the results from these cohorts, rather than linear inverse or direct relations between DEHP concentration and FSIQ, a U-shaped dose-response curve could be considered as the lowest and highest concentrations within the study range and are associated with higher neurodevelopmental scores and intermediate concentrations are associated with lower IQ. While non-monotonic associations cannot be excluded, the experimental and epidemiologic evidence of such U-shaped health effects is limited. Andrade et al. [69] showed a non-monotonic dose–response on rat brain aromatase activity in a study following in utero and lactational exposure to DEHP. Do et al. [70] presented non-monotonic dose effects of in utero exposure to DEHP on reproductive markers in male mouse fetuses. DEHP exposure has been associated with further non-monotonic neuroendocrine effects in experimental animals [71]. Finally, Choi et al. [72] observed a non-monotonous association (U-shaped) between the level of DEHP and the risk of atopic dermatitis in children. Further evidence on the specific equations that might associate phthalates and child neurodevelopment must be obtained, and currently alternatives to a linear relation in epidemiologic studies remain scientifically weak.

A second possible interpretation of these results would be that cohort-specific, residual confounding is still present in the study. Statistical analyses were conducted using harmonized data of three European existing cohorts. In the evaluation of the association between phthalate/HEXAMOLL^®^ DINCH biomarkers of exposure and child neurodevelopment, only common covariates were considered. Residual confounding might be present despite regression models were adjusted for the education level of the household, child’s sex, and BMI z-score, and many more factors were considered as potential confounders and then dismissed in the analytical phase, according to an *a priori* Directed Acyclic Graph (DAG) (Supplementary Figure S1) elaborated during the HBM4EU project [73].

According to epidemiologic principles of confounding, we propose that candidate factors acting as residual confounders in this study may be particularly dietary components that are directly associated with DEHP concentration [74] and concurrently, either directly [75] or through mediation (e.g., socioeconomic status) [76,77,78] affecting the child’s neurodevelopment. In fact, certain items in the diet might act as positive—i.e., away from the null—confounders but in the opposite direction depending on the country’s diet. In the results of our study, the two cohorts showing opposite effects of DEHP originate from a Northern (Denmark) and a Southern (Italy) European country. The Diet of Italian and Danish children differ by type of fat consumed. According to Rippin et al. [79], the proportion of the type of fat represented by saturated fats, monounsaturated fats (MUFAs), and polyunsaturated fats (PUFAs) in Italian children is 33%, 46%, and 12%; corresponding figures for Danish children being 40%, 36%, and 16%. Furthermore, the European Food Safety Authority (EFSA) [80] has estimated that children’s daily consumption of milk and dairy products in Italy and Denmark is 267 g and 500 g, respectively. Considering the distribution of DEHP by dietary item, for example, it has been reported that olive oil may be contaminated with DEHP in Italy [81]. However, a Mediterranean diet, particularly a high intake of olive oil, is also associated with a healthy lifestyle, a higher socioeconomic status [82,83,84,85], and better cognitive performance in children [75,86].

Should the null hypothesis (DEHP not associated with FSIQ) be the correct explanation the lack of inclusion of olive oil intake in the Italian model or an incomplete socioeconomic status characterization may have determined a confounded, overestimated, positive β for DEHP metabolites concentration in the Italian NAC-II. As opposed to the latter hypothesis, items in the Danish diet may have acted as a confounder leading to an underestimation of the β for DEHP metabolites in the Danish OCC; should DEHP, for example, be associated with the consumption of highly processed or packaged dairy products [74], again, potentially mediated by socioeconomic status. In this case, inverse, but confounded, β for DEHP metabolites concentration in the Danish OCC could emerge because no adjustment has been made for other molecules, nor type of fat, present in certain dairy foods that may have negative effects on neurodevelopment. The role of residual confounding as an explanation for the results of this study that are heterogeneous by country, rests on the underlying assumption that cultural and socioeconomic status determines different lifestyles, especially different dietary choices, in different countries and that DEHP exposure may be associated with opposite levels of society by country. A recent investigation on the association between the socioeconomic position and exposure to multiple environmental contaminants, phthalates included, conducted in six European mother–child cohorts has shown that the association may be present in both directions depending on the contaminant [87]. Heterogeneity of the direction by country and study area has also been reported [88,89].

Regarding possible residual confounding by variables that were included as terms in the linear regression models, it is very unlikely that the child’s sex and body mass index z-score were measured and coded incorrectly due to their easy ascertainment and calculation. The situation differs for socioeconomic status since this determinant of child’s neurodevelopment may depend on a combination of educational, financial, social, cultural, and human capital resources available at the individual and group level. A composite family affluence scale is generally preferred to describe socioeconomic status than a single variable. Due to differences by country in the three original studies, the only common available information was educational level (ISCED). Furthermore, ISCED was categorized as Low education (ISCED 0–2), Medium education (ISCED 3–4), and High education (ISCED ≥5) to avoid categories holding too few subjects in certain countries. This choice may have determined severe under-adjustment and subsequent residual confounding for socioeconomic variables not included in the model, such as the income of the household and occupation of the mother and/or father.

In addition, within cohort error in the β for DEHP may have been caused by the absence of inclusion of effect modification (i.e., synergistic) terms for child’s sex, BMI, education, or for other variables not included in the analyses. The reason for such exclusion was the relatively small size (N = 300) of each cohort.

After modeling, confounding, and effect modification, a fourth common reason determining unexpected results is measurement error in the exposure assessment. Urine phthalates in the three cohorts were measured by three different laboratories, although all these laboratories adhered to the same QA/QC and EQUAS procedures. For HEXAMOLL^®^ DINCH, OCC urine samples were analyzed in Denmark, while NAC-II and PCB cohort urine samples were in the same lab in Slovakia.

Furthermore, as in any cross-sectional study design, the simultaneous measurement of phthalate/HEXAMOLL^®^ DINCH exposure and assessment of neurodevelopment does not take into account the latency between exposure and the future effect on neurodevelopment. The phthalate and HEXAMOLL^®^ DINCH metabolite levels were not determined during in utero phase. Therefore, potential intra uterine effects were not covered by this study.

Phthalate metabolites are non-persistent chemicals with short biological half-lives in the range of hours/days. Therefore, the use of a single urine sample to characterize mid- to long-term exposure frequently might lead to exposure misclassification [90]. However, as they are taken up continuously, the levels reached might be stable according to stable consumer and nutritional habits. Future cohort studies should consider using recently validated methods to pool multiple urine samples, improving exposure characterization while maintaining cost-effectiveness [91,92].

As far as potential misclassification of the outcome variables (i.e., FSIQ of the WISC), in this analysis, WISC’s scores were calculated according to three different editions and are not directly comparable. This might be the most relevant limitation of this analysis. For this reason, we conducted separate analyses for each cohort and did not present meta-analytical results. In addition, the distributions of the FSIQ score in the three cohorts were statistically different, which may be partially explained by the different percentage distribution of the highest education level of the child household. Furthermore, the mean value of IQ score of children of the Italian cohort was 9 points higher than those of the Italian standard population. Moreover, the mean FSIQ score of children in the PCB cohort was 19 points lower than the Slovak standard population. In contrast, the mean FSIQ of the Danish cohort was only two points lower compared to the standard Danish population.

### 4.2. External Consistency

Evidence from the literature about phthalates exposure in children and their neurodevelopment does not show consistent results, either. In recent research by Hyland et al. [93], a positive association between FSIQ score (WISC 4th edition) assessed at 7-years, and the prenatal exposure to the ∑ of DEHP biomarkers was found in girls. Moreover, the general intellectual ability assessed through the Intelligence and Development Scales on 7-year-old Polish children was positively associated with prenatal oxo-MEHP exposure, whose median value corresponds to 1.3 µg/L [94].

On the contrary, Kim et al. [95] reported a robust inverse association between urinary DEHP’s biomarkers levels (OH-MEHP and oxo-MEHP geometric mean concentrations: 64.1 µg/g crt and 47.6 µg/g crt, respectively) of 6-year-old Korean children and their FSIQ score (WISC Korean 3rd edition), even after adjustment for demographic variables and Comprehensive Attention test scores (OH-MEHP β = −9.27; 95% CI: −17.25–−1.29; oxo-MEHP β = −9.83; 95% CI: −17.44–−2.21). Moreover, urinary concentrations of DEHP oxidative metabolites at 3 years of age were inversely associated with children’s FSIQ score at 5 and 8 years [96]. Higher postnatal urinary DEHP’s biomarker concentrations were associated with lower intelligence quotient scores in Taiwanese children 2–12 years of age [97]. A 2018 meta-analysis performed by Lee et al. [47] concluded that exposure to DEHP could be a risk for adequate children’s neurodevelopment.

The results on the relation between the exposure to the remaining phthalates biomarkers (MEP, MBzP, MnBP, and MiBP) and FSIQ score in the three cohorts are not convincing. In agreement with our results, the systematic review and meta-analysis of Radke et al. [98] reported that the evidence for the association between cognition and DEHP’s biomarkers, MnBP, MBzP, and MiBP is characterized by uncertainties that prevent establishing a causal conclusion in both directions.

For HEXAMOLL^®^ DINCH biomarkers, none of the current analyses showed any robust or consistent inverse associations with the child’s FSIQ score in the three cohorts. As this is the first evaluation considering HEXAMOLL^®^ DINCH biomarkers exposure in children and their neurodevelopment, further studies are needed to confirm or contradict the current findings.

One of the reasons for such overall negative, or at least unconvincing, results might be that exposure levels in these children were too low to show any effects. Indeed, urinary concentrations of phthalates and HEXAMOLL^®^ DINCH biomarkers in these Italian, Danish and Slovak children were far below the Human Biomonitoring Guidance Values [99].

## 5. Conclusions

We found contrasting evidence on the association between phthalate biomarkers and child cognitive performance in the three European children cohorts. Therefore, this analysis does not add evidence of a causal effect of phthalates exposure on a child’s IQ. Finally, our study also does not provide evidence of an association between HEXAMOLL^®^ DINCH exposure and an adverse IQ performance. Non-monotonic effects of these endocrine-disrupting chemicals could also be taken into account. The timing of exposure and the synergic effect of other substances should be the subject of further approaches in addressing this scientific hypothesis.

## Figures and Tables

**Table 1:** Child and family characteristics in the NAC-II, OCC, and PCB cohorts.

Characteristics	NAC-II	OCC	PCB Cohort
**Child’s Sex, N (%):**			
Male	150 (50.0)	165 (55.0)	133 (44.3)
Female	150 (50.0)	135 (45.0)	167 (55.7)
**Highest education level of the household of the child, N (%):**			
Low education (ISCED 0–2)	26 (8.7)	41 (13.7)	16 (5.3)
Medium education (ISCED 3–4)	139 (46.3)	154 (51.3)	222 (74.0)
High education (ISCED ≥5)	132 (44.0)	105 (35.0)	45 (15.0)
Missing	3 (1.0)	0 (0.0)	17 (5.7)
**Child’s BMI z-score, mean ± SD (N):**	0.7 ± 1.2 (275)	−0.1 ± 1.0 (294)	0.7 ± 1.3 (298)

Abbreviations: NAC-II, Northern Adriatic Cohort; OCC, Odense Child Cohort; ISCED, International Standard Classification of Education; SD, standard deviation; BMI, body mass index.

**Table 2:** Urinary concentrations of phthalates and HEXAMOLL^®^ DINCH biomarkers in the NAC-II, OCC, and PCB cohorts.

Biomarkers of Exposure	N	Geometric Mean (95% CI)	25th Percentile	Median	75th Percentile	90th Percentile
***Phthalates*** **(µg/L)**						
***MiBP:***						
NAC-II	299	29.6 (26.9–32.4)	18.6	31.9	49.8	74.1
OCC	300	12.2 (11.1–13.4)	7.2	12.4	19.2	37.4
PCB cohort	295	59.7 (53.6–66.4)	32.4	59.1	110.1	183.4
***MnBP:***						
NAC-II	297	19.0 (17.2–21.0)	11.0	19.1	32.1	57
OCC	300	12.1 (11.1–13.3)	7.2	12.7	20.0	29.6
PCB cohort	296	75.3 (68.9–82.3)	45.0	74.8	128.1	213.0
***MBzP:***						
NAC-II	299	5.6 (5.0–6.3)	2.8	5.9	10.8	21.3
OCC	297	1.3 (1.1–1.4)	0.6	1.2	2.6	5.1
PCB cohort	287	3.5 (3.0–4.1)	1.0	5.0	10.1	13.8
***MEP:***						
NAC-II	300	59.1 (52.0–67.2)	27.8	55.5	115.5	222.2
OCC	294	7.2 (6.5–7.9)	4.2	6.7	11.8	19.3
PCB cohort	295	34.6 (30.4–39.4)	14.8	30.3	67.4	154.2
***5OH-MEHP:***						
NAC-II	299	17.3 (15.8–18.9)	10.6	17.4	27.1	46.3
OCC	300	4.8 (4.3–5.3)	2.9	5.0	7.6	12.4
PCB cohort c	296	24.9 (23.0–26.9)	17.0	25.3	38.6	57.4
***5cx-MEPP:***						
NAC-II	299	21.3 (19.4–23.3)	12.4	21.7	38.3	53.1
OCC	300	6.9 (6.3–7.5)	4.3	6.6	10.8	37.4
PCB cohort	296	34.4 (31.7–37.3)	22.4	35.0	53.8	81.2
***5oxo-MEHP:***						
NAC-II	299	8.8 (8.0–9.6)	5.4	8.9	14.6	23.7
OCC	300	3.3 (3.0–3.7)	2.0	3.4	5.5	8.8
PCB cohort	296	21.5 (20.0–23.3)	15.1	21.3	32.8	46.8
***5OH-MEHP+5oxo-MEHP:***						
NAC-II	299	26.5 (24.3–28.9)	15.9	27.0	42.2	65.4
OCC	300	8.1 (7.4–8.9)	5.0	8.5	12.8	21.6
PCB cohort	296	46.8 (43.4–50.5)	31.5	47.6	70.4	102.0
***5OH-MEHP+5cx-MEPP:***						
NAC-II	299	39.5 (36.1–43.1)	23.2	40.3	64.4	95.1
OCC	300	11.8 (10.8–12.9)	7.5	11.6	18.0	28.3
PCB cohort	296	59.8 (55.3–64.7)	39.4	61.4	94.2	142.1
***HEXAMOLL^®^ DINCH*** **(µg/L)**						
***OH-MINCH:***						
NAC-II	300	3.6 (3.2–4.1)	1.8	3.3	6.1	15.8
OCC	300	3.2 (2.8–3.7)	1.5	3.0	6.5	13.0
PCB cohort	300	2.3 (2.0–2.6)	1.2	2.0	4.1	9.0
***cx-MINCH:***						
NAC-II	300	2.3 (2.0–2.6)	1.1	2.0	3.8	8.8
OCC	300	2.1 (1.9–2.4)	1.1	1.7	4.1	9.4
PCB cohort	299	1.1 (1.0–1.3)	0.5	1.1	2.0	4.4
***OH-MINCH+cx-MINCH:***						
NAC-II	300	6.0 (5.3–6.8)	3.0	5.3	9.9	25.2
OCC	300	5.5 (4.8–6.2)	2.7	4.9	10.4	22.8
PCB cohort	300	3.4 (3.0–3.9)	1.8	3.1	6.0	14.2

Abbreviations: 95% CI, 95% confidence interval; MiBP, mono-isobutyl phthalate; MnBP, mono-n-butyl phthalate; MBzP, mono-benzyl phthalate; MEP, mono-ethyl phthalate; 5OH-MEHP, mono(2-ethyl-5-hydroxy-hexyl) phthalate; 5cx-MEPP, mono(2-ethyl-5-carboxy-pentyl) phthalate; 5oxo-MEHP, mono(2-ethyl-5-oxo-hexyl) phthalate; HEXAMOLL^®^ DINCH, cyclohexane-1,2-dicarboxylic acid diisononyl ester; OH-MINCH, cyclohexane-1,2-dicarboxylate-mono-(7-hydroxy-4-methyl)octyl ester; cx-MINCH, cyclohexane-1,2-dicarboxylate-mono-(7-carboxylate-4-methyl)heptyl ester.

**Table 3:** Distribution (mean ± SD) of FSIQ score by potential confounders in the NAC-II, OCC, and PCB cohorts.

	FSIQ Score					
Characteristics	NAC-II	*p*-Value	OCC	*p*-Value	PCB Cohort	*p*-Value
**Highest education level of the household of child, mean ± SD:**						
Low education (ISCED 0–2)	103 ± 10	<0.01 ^a^	95 ± 14	0.19 ^a^	56 ± 9	<0.01 ^a^
Medium education (ISCED 3–4)	108 ± 11		98 ± 12		81 ± 13	
High education (ISCED ≥5)	112 ± 10		100 ± 11		91 ± 16	
**Child’s sex, mean ± SD:**						
Male	109 ± 11	0.77 ^a^	97 ± 12	0.06 ^a^	79 ± 16	0.09 ^a^
Female	109 ± 10		100 ± 12		82 ± 15	

^a^ ANOVA was applied.

## Data Availability

Metadata of the three cohorts are available in IPCHEM, the European Commission’s Information Platform for Chemical Monitoring. The summary statistics (percentiles P5, P10, P25, P50, P75, P90, P95) of the exposure biomarkers will be available in the openly accessible online European HBM dashboard (https://www.hbm4eu.eu/eu-hbm-dashboard/ accessed on 30 June 2022) and IPCHEM (https://ipchem.jrc.ec.europa.eu/ accessed on 30 June 2022), where it can be accessed. Data sharing of individual-level data is possible upon request.

## Supplementary Materials

The following supporting information can be downloaded at: https://www.mdpi.com/article/10.3390/toxics10090538/s1, Table S1: List of scores (variables) from WISC-III, WISC-IV and WISC-V; Table S2: Urinary concentrations of phthalates and HEXAMOLL^®^ DINCH biomarkers standardized for creatinine and creatinine in the NAC-II, OCC, and PCB cohorts; Table S3: Multilevel fixed-effect linear regression models for the FSIQ with cohort-level predictors and natural logarithm transformation of phthalates/HEXAMOLL^®^ DINCH biomarkers standardized for creatinine. (N cohorts = 3); Table S4: Simple and multiple linear regression models between the FSIQ score and the natural logarithm transformation of phthalates/DINCH biomarkers standardized for creatinine in the NACI-II, OCC, and PCB cohort; Table S5: Simple and multiple linear regression models between the FSIQ score and the natural logarithm transformation of phthalate/DINCH biomarkers in the NAC-II, OCC, and PCB cohort; Table S6: Simple and multiple linear regression models between the FSIQ score and the natural logarithm transformation of the sum of phthalate/DINCH biomarkers expressed in molar unit standardized for creatinine in the NAC-II, OCC, and PCB cohort; Figure S1: Directed acyclic graph (DAG) representing the assumed relationships between the exposure phthalates and HEXAMOLL^®^ DINCH and the outcome neurodevelopment, specific for children; Figure S2: Forest plot showing the association between FSIQ score and the natural logarithm of phthalates/ HEXAMOLL^®^ DINCH biomarkers standardized for creatinine adjusted for the highest education level of the household of the child, child’s sex and body mass index z-score in the NAC-II; Figure S3: Forest plot showing the association between FSIQ score and the natural logarithm of phthalates/ HEXAMOLL^®^ DINCH biomarkers standardized for creatinine adjusted for the highest education level of the household of the child, child’s sex and body mass index z-score in the OCC; Figure S4: Forest plot showing the association between FSIQ score and the natural logarithm of phthalates/ HEXAMOLL^®^ DINCH biomarkers standardized for creatinine adjusted for the highest education level of the household of the child, child’s sex and body mass index z-score in the PCB cohort.
