# Supplementary material for: Concurrent Assessment of Phthalates/HEXAMOLL® DINCH Exposure and Wechsler Intelligence Scale for Children Performance in Three European Cohorts of the HBM4EU Aligned Studies
Source: Toxics. 2022 Sep 16;10(9):538. doi: 10.3390/toxics10090538 (PMC9502751; doi:10.3390/toxics10090538)
Supplement: Supplementary file 1 [file toxics-10-00538-s001.zip › toxics-1873053-SI.pdf]

Supplementary Materials

Phthalates/HEXAMOLL® DINCH Exposure and Wechsler Intelligence Scale for Children Performance in Three European Cohorts of the HBM4EU Aligned Studies

Valentina Rosolen, Elisa Giordani, Marika Mariuz, Maria Parpinel, Luca Ronfani, Liza Vecchi Brumatti, Maura Bin, Gemma Calamandrei, Vicente Mustieles, Liese Gilles, Eva Govarts, Kirsten Baken, Laura Rodriguez Martin, Greet Schoeters, Ovnair Sepai, Eva Sovcikova, Lucia Fabelova, Miroslava Šidlovská, Branislav Kolena, Tina Kold Jensen, Hanne Frederiksen, Marike Kolossa-Gehring, Rosa Lange, Petra Apel, Argelia Castano, Marta Esteban López, Griet Jacobs, Stefan Voorspoels, Helena Jurdáková, Renáta Górová and Fabio Barbone

Description of WISC-III, WISC-IV, and WISC-V.

The three editions of the Wechsler Intelligence Scale of Children (WISC) test assess and measure different aspects of the child's neurodevelopment. The core subtest and the index scores of each edition are listed in Table S1

Table S1. List of scores (variables) from WISC-III, WISC-IV and WISC-V.

**WISC-III**		**WISC-IV**		**WISC-V**	
**CORE SUBTEST**	**Min-Max**	**CORE SUBTEST**	**Min-Max**	**CORE SUBTEST**	**Min-Max**
Similarities	1-19	Similarities	1-19	Similarities	1-19
Comprehension	1-19	Comprehension	1-19		1-19
Vocabulary	1-19	Vocabulary	1-19	Vocabulary	1-19
Arithmetic	1-19				1-19
Information	1-19				
Block Design	1-19	Block Design	1-19	Block Design	1-19
Picture Completion	1-19	Matrix Reasoning	1-19	Visual Puzzles	1-19
Coding	1-19	Picture Concept	1-19		
Object Assembly	1-19				
Picture Arrangement	1-19				
		Digit Span	1-19	Digit Span	1-19
		Letter-Number Seq	1-19	Picture Span	1-19
		Coding	1-19	Coding	1-19
		Symbol Search	1-19	Symbol Search	1-19
				Matrix reasoning	1-19
				Figure Weights	1-19
INDEX SCORES	Min-Max	INDEX SCORES	Min-Max	INDEX SCORES	Min-Max
Verbal IQ (VIQ)	40-160	Verbal Comprehension Index (VCI)	40-160	Verbal Comprehension Index (VCI)	40-160
Performance IQ (PIQ)	40-160	Perceptual Reasoning Index (PRI)	40-160	Visual Spatial Index (VSI)	40-160
				Fluid Reasoning Index (FRI)	40-160
		Working Memory Index (WMI)	40-160	Working Memory Index (WMI)	40-160
		Processing Speed Index (PSI)	40-160	Processing Speed Index (PSI)	40-160
Full Scale IQ (FSIQ)	40-160	Full Scale IQ (FSIQ)	40-160	Full Scale IQ (FSIQ)	40-160

The WISC-III edition[1,2] comprises 10 core subtests that compute Verbal IQ (VIQ) and Performance IQ (PIQ), which, when combined, yield the Full-Scale IQ (FSIQ) (Mean = 100 and Standard Deviation = 15). The Verbal scale includes Vocabulary, Similarities, Comprehension, Arithmetic, and Digit Span subtests, whereas the performance scale includes Block Design, Picture Arrangement, Picture Completion, Object Assembly, and Coding subtests for all subtests (mean = 10 and standard deviation = 3).

The WISC-IV[1,3] comprises 10 core subtests, yielding four index scores that combine into the FSIQ. The Verbal Comprehension Index (VCI) is similar to the previous VIQ and consists of three of the previous five 3rd edition subtests (Similarities, Comprehension, and Vocabulary). The Perceptual Reasoning Index (PRI) presents more changes when compared to the previous PIQ. Specifically, the PRI still comprises Block Design but adds two new subtests, Matrix Reasoning and Picture Concepts. Only one of these tasks is timed and implies the use of materials (Block Design) compared to four subtests of the WISC-III PIQ.
The Working Memory Index (WMI; which includes Digit Span and Letter-Number Sequencing) and the Processing Speed Index (PSI; that includes Coding and Symbol Search) now represent distinct indexes, as well as being part of the FSIQ. Index standard scores have a mean of 100 and a standard deviation of 15.

The WISC-V[4] comprises 10 core subtests, yielding five index scores that combine into the FSIQ. The Verbal Comprehension Index (VCI), the Visual Spatial Index (VSI), the Fluid Reasoning Index (FRI), the Working Memory Index (WMI), and the Processing Speed Index (PSI). The FSIQ is derived from 7 of the 10 primary subtests:

- the Verbal Comprehension Index is derived from the Similarities and Vocabulary subtests;
- the Visual Spatial Index (VSI) is derived from the Block Design and Visual Puzzles subtests;
- the Fluid Reasoning Index (FRI) is derived from the Matrix Reasoning and Figure Weights subtests;
- the Working Memory Index is derived from the Digit Span and Picture Span subtests;
- the Processing Speed Index is derived from the Coding and Symbol Search subtests;
- Index standard scores have a mean of 100 and a standard deviation of 15.

Computation of the Core Subtests and Index Scores

The first step is to sum the individual item scores to obtain the raw scores according to the instructions present in the WISC manual. To obtain the core subtests, the sum of raw scores are weighted for the child's age at which the test was administered using the conversion tables (present in the manual of the WISC), which are different for each country and in each WISC edition. The second step is to sum the core subtests, according to the instructions present in the WISC manual, and transform these sums into intelligence quotient based on the population-based reference data (standard population) with a mean of 100 and standard deviation of 15. Each WISC edition in each country has an excellent standardization sample (more than 2000 children ages 6:0 to 16:11) that closely represents the population of each country on stratification variables of race, ethnicity, geographic region, parent education level, and gender.

1. Nader AM, Jelenic P, Soulières I. Discrepancy between WISC-III and WISC-IV Cognitive Profile in Autism Spectrum: What Does It Reveal about Autistic Cognition?. PLoS One. 2015;10(12):e0144645. Published 2015 Dec 16. doi:10.1371/journal.pone.0144645
2. Wechsler D. Wechsler Intelligence Scale for Children- Third Edition: Canadian (WISC-III). Psychological Corporation. ed. Toronto, Canada1991.
3. Wechsler D. Wechsler Intelligence Scale for Children- Fourth Edition: Canadian (WISC-IV). Psychological Corporation. ed. Toronto, Canada2005.
4. Olivier T.W., Mahone E.M., Jacobson L.A. (2018) Wechsler Intelligence Scale for Children. In: Kreutzer J.S., DeLuca J., Caplan B. (eds) Encyclopedia of Clinical Neuropsychology. Springer, Cham. https://doi.org/10.1007/978-3-319-57111-9_1605

Table S2. Urinary concentrations of phthalates and HEXAMOLL® DINCH biomarkers standardized for creatinine and creatinine in the NAC-II, OCC, and PCB cohorts.

Biomarker of Exposure	N	Geometric Mean (95%CI)	25th Percentile	Median	75th Percentile	90th Percentile
Phthalates (µg/g crt)						
MiBP:						
NAC-II	299	35.3 (32.9–37.9)	24.6	34.2	50.2	73.4
OCC	300	17.7 (16.3–19.3)	12.0	16.1	26.8	45.5
PCB cohort	294	49.6 (44.1–55.7)	26.2	48.4	81.0	154.5
MnBP:						
NAC-II	297	22.8 (21.0–24.7)	14.6	21.8	33.0	54.1
OCC	300	17.7 (16.4–19.0)	11.8	11.8	25.6	36.5
PCB cohort	295	62.4 (56.2–69.2)	35.8	59.0	102.8	168.1
MBzP:						
NAC-II	299	6.7 (6.1–7.4)	3.9	6.7	11.1	21.7
OCC	297	1.8 (1.6–2.1)	0.9	1.6	3.0	7.1
PCB cohort	286	2.9 (2.5–3.4)	0.9	4.0	7.6	13.4
MEP:						
NAC-II	300	70.3 (62.7–78.9)	33.2	63.5	131.9	257.7
OCC	294	10.3 (9.4–11.3)	6.0	9.0	15.0	25.6
PCB cohort	294	28.7 (24.9–33.0)	13.2	24.0	54.9	141.3
5OH-MEHP:						
NAC-II	299	20.7 (19.2–22.3)	14.3	19.2	28.8	45.3
OCC	300	6.9 (6.3–7.6)	4.5	6.6	11.1	16.7
PCB cohort	295	20.6 (18.8–22.6)	14.0	20.3	31.8	47.2
5cx-MEPP:						
NAC-II	299	25.4 (23.6–27.3)	16.8	24.2	37.3	54.5
OCC	300	10.0 (9.2–10.8)	6.3	9.4	14.3	24.8
PCB cohort	295	28.5 (25.9–31.3)	18.0	28.2	46.7	68.3
5oxo-MEHP:						
NAC-II	299	10.5 (9.7–11.3)	6.9	9.8	15.0	22.5
OCC	300	4.5 (4.4–5.3)	2.9	4.6	7.5	13.8
PCB cohort	295	17.8 (16.3–19.6)	12.1	18.1	25.6	40.9
5OH-MEHP+5oxo-MEHP:						
NAC-II	299	31.6 (29.4–33.9)	21.3	29.6	44.6	66.8
OCC	300	11.8 (10.8–12.9)	7.7	11.1	18.7	32.9
PCB cohort	295	38.8 (35.4–42.5)	27.1	39.1	57.7	87.2
5OH-MEHP+5cx-MEPP:						
NAC-II	299	47.1 (43.9–50.5)	32.4	43.5	68.1	102.1
OCC	300	17.1 (15.8–18.6)	10.8	16.2	24.9	46.2
PCB cohort	295	49.5 (45.2–54.3)	32.1	48.0	76.6	111.4
HEXAMOLL® DINCH (µg/g crt)						
OH-MINCH						
NAC-II	300	4.3 (3.8–4.9)	2.0	3.6	6.7	19.7
OCC	300	4.7 (4.1–5.3)	2.2	4.0	9.1	21.1
PCB cohort	299	1.9 (1.7–2.1)	0.9	1.6	3.3	6.9
cx-MINCH						
NAC-II	300	2.7 (2.4–3.1)	1.2	2.2	4.1	11.1
OCC	300	3.1 (2.7–3.5)	1.5	2.8	5.5	13.6
PCB cohort	298	0.9 (0.8–1.0)	0.5	0.9	1.6	2.9
OH-MINCH+cx-MINCH						
NAC-II	300	7.1 (6.3–8.0)	3.4	6.2	10.5	30.6

OCC	300	7.9 (7.1–9.0)	3.9	6.9	15.3	34.3
PCB cohort	299	2.8 (2.5–3.2)	1.4	2.5	5.0	10.1
***Crt* (g/L)**						
NAC II	300	0.8 (0.8–0.9)	0.6	0.9	1.2	1.5
OCC	300	0.7 (0.6–0.7)	0.5	0.7	1.0	1.1
PCB cohort	299	1.2 (1.1–1.3)	0.9	1.3	1.8	2.3

Abbreviations: NAC-II, Northern Adriatic cohort II; OCC, Odense Child cohort; crt, creatinine; 95%CI, 95% confidence interval; MiBP, mono-isobutyl phthalate; MnBP, mono-n-butyl phthalate; MBzP, mono-benzyl phthalate; MEP, mono-ethyl phthalate; 5OH-MEHP, mono(2-ethyl-5-hydroxy- hexyl) phthalate; 5cx-MEPP, mono(2-ethyl-5-carboxy- pentyl) phthalate; 5oxo-MEHP, mono(2-ethyl-5-oxo-hexyl) phthalate; HEXAMOLL® DINCH, cyclohexane-1,2-dicarboxylic acid diisononyl ester; OH-MINCH, cyclohexane-1,2-dicarboxylate-mono-(7-hydroxy-4-methyl)octyl ester; cx-MINCH, cyclohexane-1,2-dicarboxylate-mono-(7-carboxylate-4-methyl)heptyl ester.

The results of the multilevel fixed-effect linear regression models are shown in Table S3. N significant association between FSIQ and the natural logarithm of any biomarker, standardized for creatinine and adjusted for potential confounders, was found. The ICC ranged between 0.45–0.47, indicating that 45–47% of the variability in FSIQ is accounted for by the cohort to which the children belong.

Table S3. Multilevel fixed-effect linear regression models for the FSIQ with cohort-level predictors and natural logarithm transformation of phthalates/HEXAMOLL® DINCH biomarkers standardized for creatinine. (N cohorts = 3).

**Biomarker of Exposure**	**Multilevel Fixed-effect Linear Regression**		
	**N**	**β (95%CI) †**	**ICC**
Phthalates (μg/g crt)			
MiBP	813	-0.21 (-1.25-0.83)	0.46
MnBP	812	0.01 (-1.11-1.12)	0.46
MBzP	803	0.38 (-0.42-1.09)	0.45
MEP	808	-0.51 (-1.32-0.30)	0.47
5OH-MEHP	814	0.27 (-0.85-1.39)	0.46
5cx-MEPP	814	0.08 (-1.07-1.22)	0.46
5oxo-MEHP	814	0.01 (-1.12-1.13)	0.46
5OH-MEHP+5oxo-MEHP	814	0.19 (-0.95-1.33)	0.46
5OH-MEHP+5cx-MEPP	814	0.20 (-0.97-1.36)	0.46
HEXAMOLL® DINCH (μg/g crt)			
OH-MINCH	817	0.68 (-0.10-1.47)	0.45
cx-MINCH	816	0.65 (-0.14-1.44)	0.45
OH-MINCH+cx-MINCH	817	0.71 (-0.09-1.50)	0.45

Abbreviations: FSIQ, Full Scale Intelligence Quotient; β, beta coefficient. †Adjusted for children's sex and body mass index z-score and the highest level of education of the household of the child. ICC, intraclass correlation coefficient.

Table S4. Simple and multiple linear regression models between the FSIQ score and the natural logarithm transformation of phthalates/ HEXAMOLL® DINCH biomarkers standardized for creatinine in the NACI-II, OCC, and PCB cohorts.

Biomarker of Exposure	NAC-II		OCC		PCB Cohort	
	**Simple Regression β (95%CI); N**	**Multiple Regression† β (95%CI); N**	**Simple Regression β (95%CI); N**	**Multiple Regression† β (95%CI); N**	**Simple Regression β (95%CI); N**	**Multiple Regression† β (95%CI); N**
Phthalates (µg/g crt)						
MiBP	0.12 (-1.90–2.13); 297	-0.05 (-2.03–1.93); 270	-0.29 (-2.22–1.65); 273	-0.45 (-2.39–1.49); 268	-0.38 (-2.15–1.38); 291	-0.06 (-1.58–1.46); 275
MnBP	-0.54 (-2.35–1.27); 295	-0.37 (-2.30–1.56); 268	-0.21 (-2.35–1.94); 273	-0.41 (-2.54–1.73); 268	0.30 (-1.67–2.28); 292	0.33 (-1.37–2.02); 276
MBzP	0.02 (-1.42–1.46); 297	0.07 (-1.35–1.48); 270	-0.21 (-1.60–1.18); 270	-0.09 (-1.48–1.29); 265	0.68 (-0.64–1.99); 283	0.61 (-0.51–1.73); 268
MEP	-0.86 (-2.08–0.36); 298	-0.13 (-1.35–1.09); 270	-0.86 (-2.08–0.36): 298	-1.21 (-2.98–0.56); 268	-0.11 (-1.57–1.35); 291	-0.77 (-2.02–0.49); 275
5OH-MEHP	2.28 (0.42–4.15); 297**	2.58 (0.65–4.51); 270**	-1.45 (-3.24–0.34); 273	-1.51 (-3.29–0.27); 268*	0.37 (-1.87–2.61); 292	0.51 (-1.42–2.44); 276
5cx-MEPP	1.70 (-0.23–3.62); 297*	1.88 (-0.04–3.80); 270*	-1.54 (-3.49–0.41); 273	-1.49 (-3.43–0.45); 268	-0.11 (-2.29–2.08); 292	0.11 (-1.77–1.99); 276
5oxo-MEHP	1.81 (-0.13–3.75); 297*	1.83 (-0.11–3.78); 270*	-1.49 (-3.30–0.33); 273	-1.53 (-3.33–0.27); 268*	-0.08 (-2.03–2.14); 292	0.28 (-1.63–2.19); 276
5OH-MEHP+5oxo-MEHP	2.32 (0.38–4.25); 297**	2.56 (0.58–4.55); 270**	-1.47 (-3.28–0.34); 273	-1.53 (-3.32–0.27); 268*	0.14 (-2.13–2.40); 292	0.39 (-1.55–2.34); 276
5OH-MEHP+5cx-MEPP	2.19 (0.21–4.17); 297**	2.48 (0.47–4.49); 270**	-1.58 (-3.51–0.35); 273	-1.58 (-3.50–0.35); 268	0.09 (-2.16–2.33); 292	0.26 (-1.68–2.19); 276
HEXAMOLL® DINCH (µg/g crt)						
OH-MINCH	0.58 (-0.58–1.74); 298	0.56 (-0.60–1.72); 270	0.42 (-0.92–1.76); 273	0.10 (-1.24–1.44); 268	1.64 (-0.02–3.30); 296**	1.05 (-0.42–2.51); 279
cx-MINCH	0.51 (-0.63–1.65); 298	0.40 (-0.74–1.53); 270	0.33 (-1.02–1.69); 273	0.13 (-1.22–1.48); 268	1.65 (-0.07–3.36); 295*	1.01 (-0.49–2.52); 278
OH-MINCH+cx-MINCH	0.56 (-0.60–1.73); 298	0.51 (-0.65–1.68); 270	0.41 (-0.97–1.79); 273	0.13 (-1.25–1.51); 268	1.70 (0.01–3.39); 296**	1.07 (-0.42–2.56); 279

†Adjusted for children's sex and body mass index z-score and the highest level of education of the household of the child. ***p*-value ≤ 0.05; **p*-value ≤ 0.10.

Table S5. Simple and multiple linear regression models between the FSIQ score and the natural logarithm transformation of phthalate/ HEXAMOLL® DINCH biomarkers in the NAC-II, OCC, and PCB cohorts.

	NAC-II		OCC		PCB Cohort	
**Biomarker of Exposure**	**Simple Regression β (95%CI); N**	**Multiple Regression† β (95%CI); N**	**Simple Regression β (95%CI); N**	**Multiple Regression† β (95%CI); N**	**Simple Regression β (95%CI); N**	**Multiple Regression† β (95%CI); N**
Phthalates (μg/L)						
MiBP	-0.09 (-1.62–1.43); 297	-0.45 (-1.96–1.06); 270	-0.16 (-1.86–1.54); 273	-0.23 (-1.92–1.46); 268	0.28 (-1.64–2.20); 292	0.28 (-1.39–1.95); 276
MnBP	-0.43 (-1.86–1.00); 295	-0.58 (-2.08–0.92); 268	-0.08 (-1.89–1.73); 273	-0.16 (-1.97–1.64); 268	1.43 (-0.86–3.72); 293	0.92 (-1.06–2.90); 277
MBzP	-0.09 (-1.3–0.89); 297	-0.22 (-1.43–0.99); 270	-0.17 (-1.48–1.15); 270	-0.02 (-1.32–1.30); 265	0.99 (-0.36–2.35); 284	0.66 (-0.50–1.82); 269
MEP	-0.78 (-1.88–0.32); 298	-0.33 (-1.43–0.77); 270	-1.13 (-2.82–0.55); 268	-1.05 (-2.75–0.64); 263	0.35 (-1.22–1.92); 292	-0.67 (-2.04–0.69); 276
5OH-MEHP	1.49 (-0.11–3.09); 297*	1.37 (-0.29–3.04); 270*	-1.18 (-2.83–0.48); 273	-1.18 (-2.82–0.46); 268	1.75 (-0.83–4.32); 293	1.26 (-0.98–3.50); 277
5cx-MEPP	0.89 (-0.63–2.40); 297	0.76 (-0.78–2.30); 270	-1.28 (-3.10–0.55); 273	-1.17 (-2.99–0.64); 268	1.06 (-1.44–3.56); 293	0.70 (-1.47–2.88); 277
5oxo-MEHP	0.99 (-0.57–2.55); 297	0.74 (-0.84–2.31); 270	-1.18 (-2.84–0.48); 273	-1.17 (-2.81–0.48); 268	1.21 (-1.44–3.87); 293	1.00 (-1.30–3.30): 277
5OH-MEHP+5oxo-MEHP	1.43 (-0.19–3.06); 297*	1.27 (-0.39–2.95); 270	-1.18 (-2.85–0.48); 273	-1.18 (-2.83–0.47); 268	1.54 (-1.13–4.21); 293	1.24 (-1.09–3.57); 277
5OH-MEHP+5cx-MEPP	1.26 (-0.35–2.87); 297	1.14 (-0.51–2.80); 270	-1.29 (-3.09–0.50); 273	-1.23 (-3.01–0.55); 268	1.40 (-1.19–3.99); 293	0.93 (-1.31–3.18); 277
HEXAMOLL® DINCH (μg/L)						
OH-MINCH	0.47 (-0.66–1.60); 298	0.30 (-0.83–1.43); 270	0.43 (-0.87–1.73); 273	0.16 (-1.14–1.46); 268	2.12 (0.49–3.75); 297**	1.28 (-0.17–2.74); 280*
cx-MINCH	0.41 (-0.70–1.52); 298	0.15 (-0.96–1.27); 270	0.35 (-0.97–1.67); 273	0.18 (-1.12–1.50); 268	2.17 (0.47–3.87); 296**	1.27 (-0.25–2.80); 279*
OH-MINCH+cx-MINCH	0.46 (-0.68–1.59); 298	0.25 (-0.88–1.39); 270	0.42 (-0.92–1.76); 273	0.19 (-1.15–1.52); 268	2.20 (0.54–3.86); 297**	1.31 (-0.17–2.80); 280*

† Adjusted for children's sex and body mass index z-score and the highest level of education of the household of the child. ***p*-value ≤0.05; **p*-value ≤0.10.

Table S6. Simple and multiple linear regression models between the FSIQ score and the natural logarithm transformation of the sum of phthalate/ HEXAMOLL® DINCH biomarkers expressed in molar unit standardized for creatinine in the NAC-II, OCC, and PCB cohorts.

	NAC-II		OCC		PCB Cohort	
**Biomarker of Exposure**	**Simple Regression β (95%CI); N**	**Multiple Regression† β (95%CI); N**	**Simple Regression β (95%CI); N**	**Multiple Regression† β (95%CI); N**	**Simple Regression β (95%CI); N**	**Multiple Regression† β (95%CI); N**
Phthalates (μmol/g crt)						
5OH-MEHP+5oxo-MEHP	2.32 (0.38-4.25); 297**	2.56 (0.58-4.55); 270**	-1.47 (-3.28-0.37); 273	-1.53 (-3.32-0.27); 268	0.14 (-2.13-2.40); 292	0.39 (-1.56-2.34); 276
5OH-MEHP+5cx-MEPP	2.19 (0.21-4.17); 297**	2.49 (0.47-4.50) ; 270**	-1.58 (-3.51-0.35); 273	-1.58 (-3.50-0.34); 268	0.09 (-2.16-2.34); 292	0.26 (-1.67-2.20); 276
HEXAMOLL® DINCH (μmol/g crt)						
OH-MINCH+cx-MINCH	0.56 (-0.60-1.73); 298	0.51 (-0.65-1.68); 270	0.41 (-0.97-1.79); 273	0.13 (-1.25-1.51); 268	1.70 (0.01-3.39); 296**	1.07 (-0.42-2.56); 279

† Adjusted for children's sex and body mass index z-score and the highest level of education of the household of the child. ***p*-value ≤0.05; **p*-value ≤ 0.10.

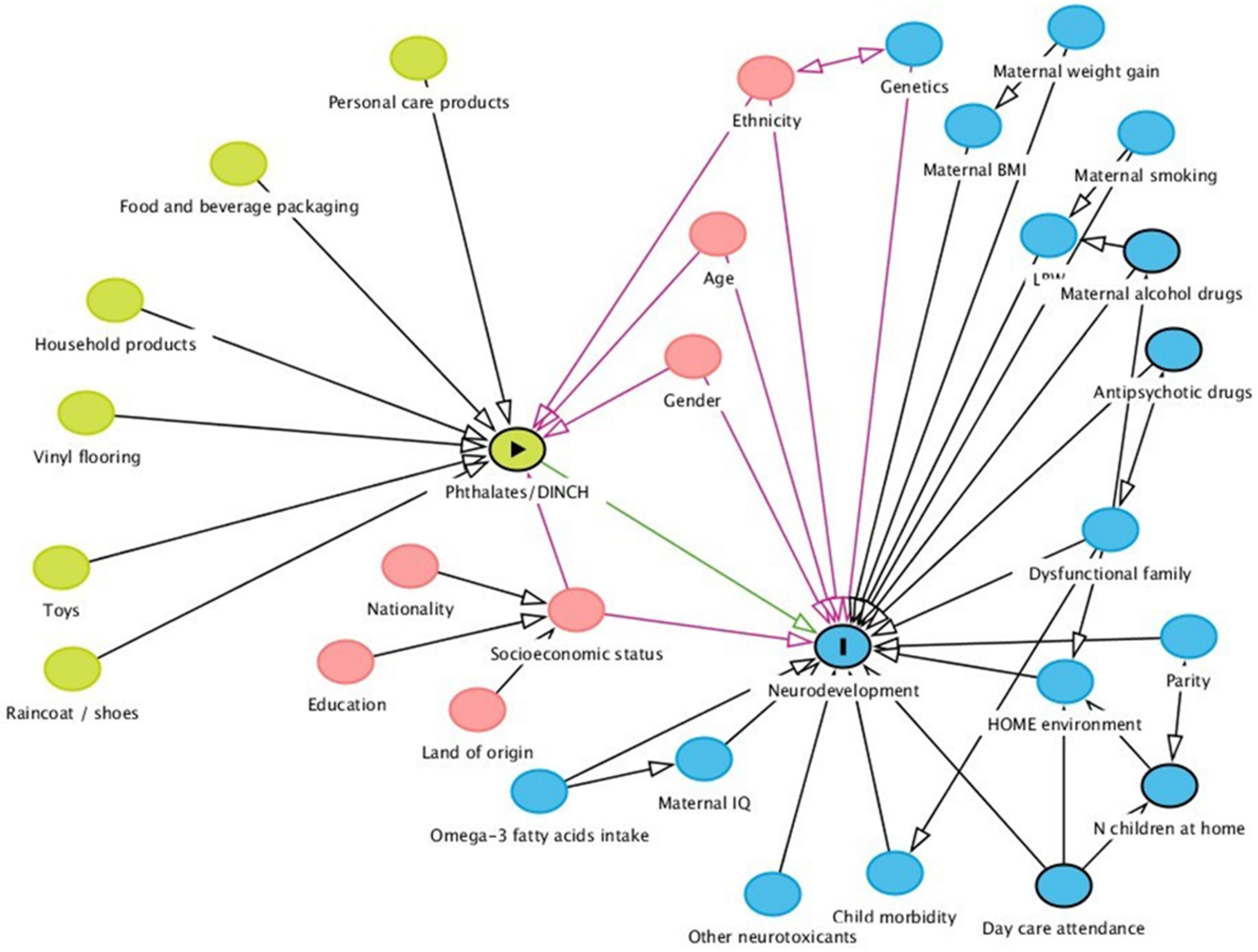

Figure S1. Directed acyclic graph (DAG) representing the assumed relationships between the exposure to phthalates and HEXAMOLL® DINCH and the outcome neurodevelopment, specific for children.

Blue nodes represent the outcome and ancestors of the outcome; green nodes the exposure and ancestors of the exposure; red nodes ancestors of both the exposure and the outcome. The green edge (= arrow) shows the assumed causal path between the exposure and outcome

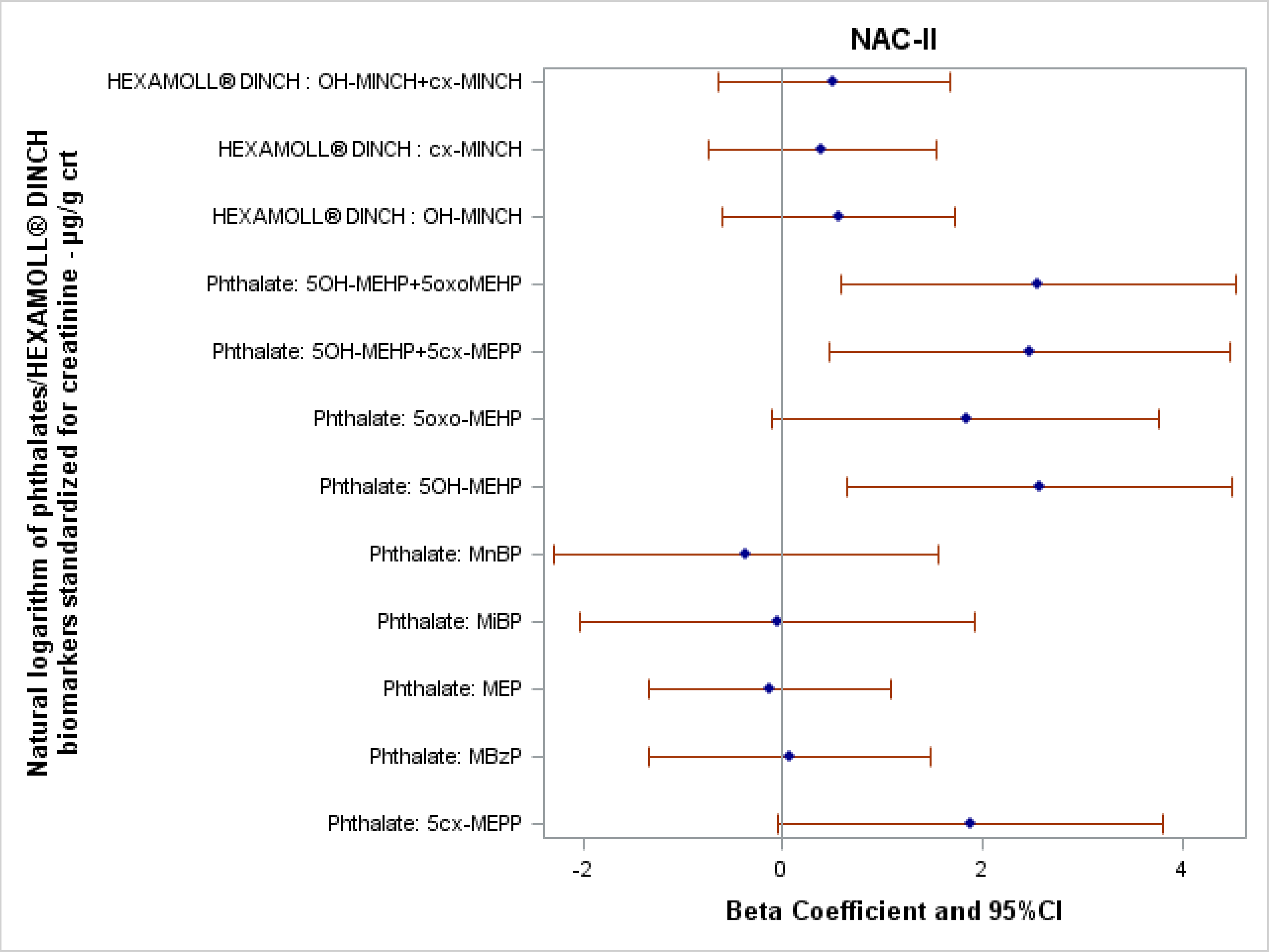

Figure S2. Forest plot showing the association between FSIQ score and the natural logarithm of phthalates/HEXAMOLL® DINCH biomarkers standardized for creatinine adjusted for the highest education level of the household of the child, child's sex, and body mass index z-score in the NAC-II.

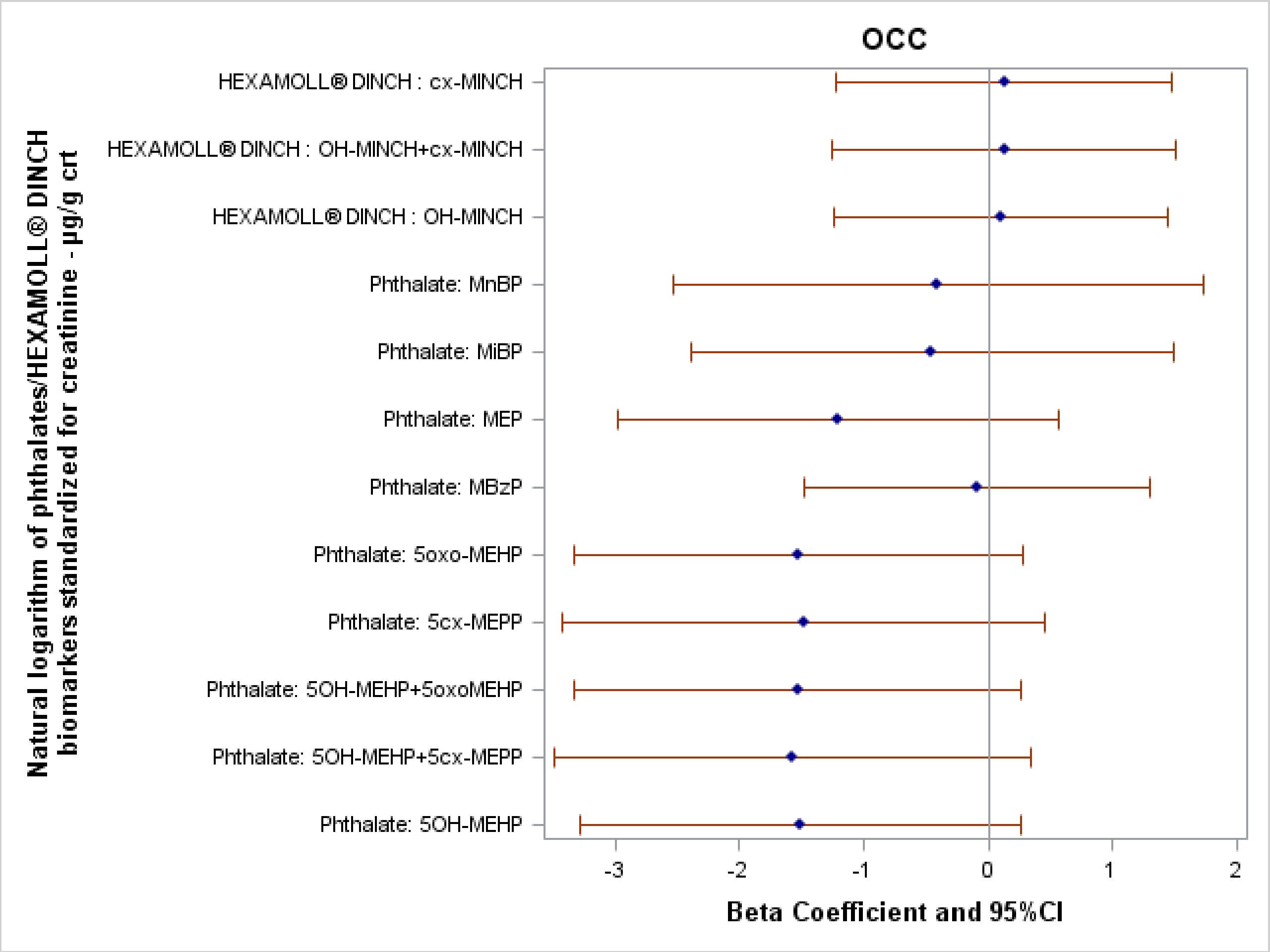

Figure S3. Forest plot showing the association between FSIQ score and the natural logarithm of phthalates/HEXAMOLL® DINCH biomarkers standardized for creatinine adjusted for the highest education level of the household of the child, child's sex, and body mass index z-score in the OCC.

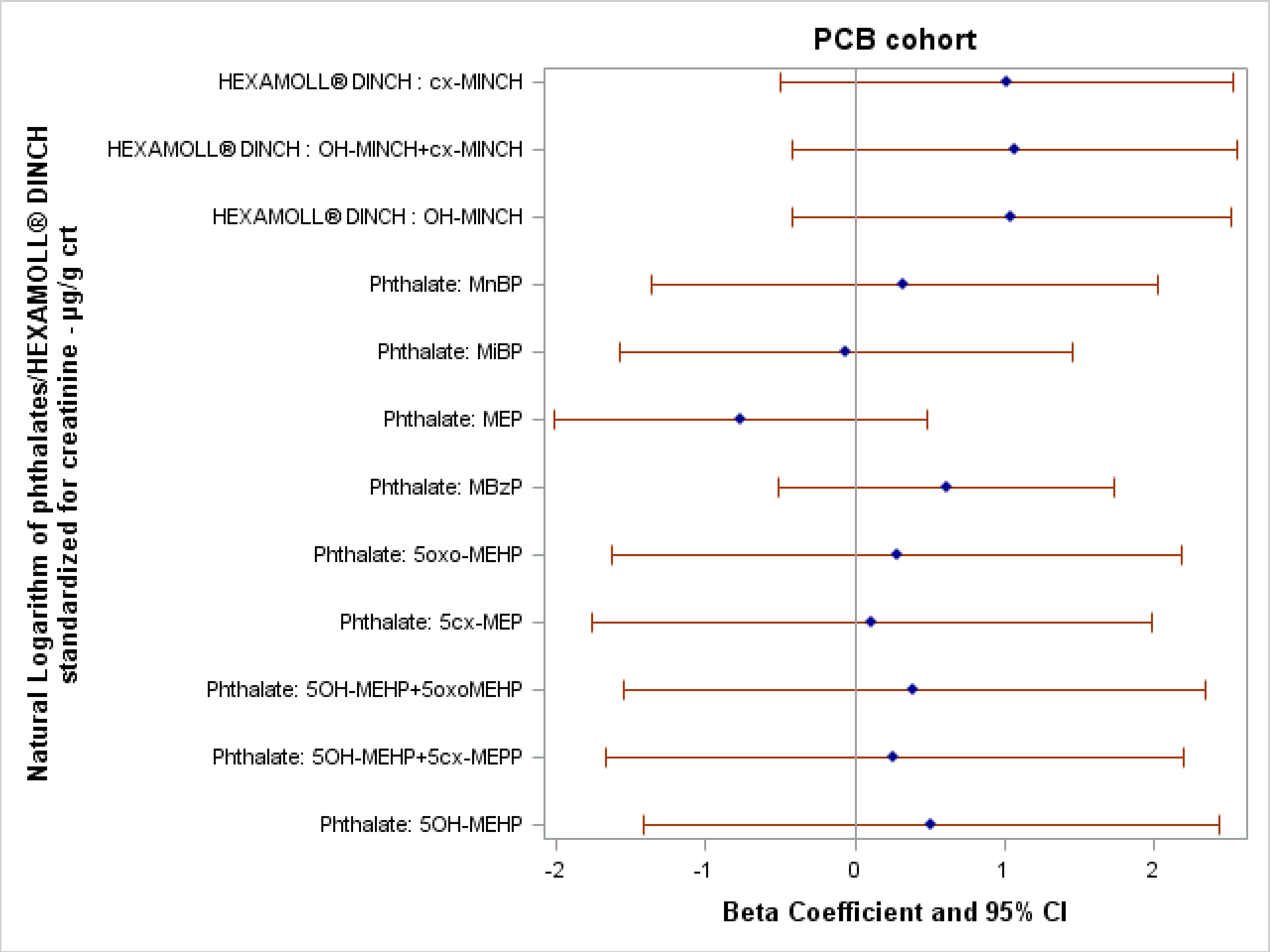

Figure S4. Forest plot showing the association between FSIQ score and the natural logarithm of phthalates/HEXAMOLL® DINCH biomarkers standardized for creatinine adjusted for the highest education level of the household of the child, child's sex, and body mass index z-score in the PCB cohort.
